# Exploration of Using Antisense Peptide Nucleic Acid (PNA)-cell Penetrating Peptide (CPP) as a Novel Bactericide against Fire Blight Pathogen *Erwinia amylovora*

**DOI:** 10.3389/fmicb.2017.00687

**Published:** 2017-04-19

**Authors:** Ravi R. Patel, George W. Sundin, Ching-Hong Yang, Jie Wang, Regan B. Huntley, Xiaochen Yuan, Quan Zeng

**Affiliations:** ^1^Department of Plant Pathology and Ecology, The Connecticut Agricultural Experiment Station, New HavenCT, USA; ^2^Department of Plant, Soil, and Microbial Sciences, Michigan State University, East LansingMI, USA; ^3^Department of Biological Sciences, University of Wisconsin–Milwaukee, MilwaukeeWI, USA; ^4^Department of Plant Biology, Michigan State University, East LansingMI, USA

**Keywords:** antisense antimicrobials, fire blight, plant disease management, peptide nucleic acid, cell penetrating peptide, *Erwinia amylovora*

## Abstract

*Erwinia amylovora* is a Gram-negative bacterial plant pathogen in the family *Enterobacteriaceae* and is the causal agent of fire blight, a devastating disease of apple and pear. Fire blight is traditionally managed by the application of the antibiotic streptomycin during bloom, but this strategy has been challenged by the development and spread of streptomycin resistance. Thus, there is an urgent need for effective, specific, and sustainable control alternatives for fire blight. Antisense antimicrobials are oligomers of nucleic acid homologs with antisense sequence of essential genes in bacteria. The binding of these molecules to the mRNA of essential genes can result in translational repression and antimicrobial effect. Here, we explored the possibility of developing antisense antimicrobials against *E. amylovora* and using these compounds in fire blight control. We determined that a 10-nucleotide oligomer of peptide nucleic acid (PNA) targeting the start codon region of an essential gene *acpP* is able to cause complete growth inhibition of *E. amylovora*. We found that conjugation of cell penetrating peptide (CPP) to PNA is essential for the antimicrobial effect, with CPP1 [(KFF)3K] being the most effective against *E. amylovora*. The minimal inhibitory concentration (MIC) of anti-*acpP*-CPP1 (2.5 μM) is comparable to the MIC of streptomycin (2 μM). Examination of the antimicrobial mechanisms demonstrated that anti-*acpP*-CPP1 caused dose-dependent reduction of *acpP* mRNA in *E. amylovora* upon treatment and resulted in cell death (bactericidal effect). Anti-*acpP*-CPP1 (100 μM) is able to effectively limit the pathogen growth on stigmas of apple flowers, although less effective than streptomycin. Finally, unlike streptomycin that does not display any specificity in inhibiting pathogen growth, anti-*acpP*-CPP1 has more specific antimicrobial effect against *E. amylovora*. In summary, we demonstrated that PNA–CPP can cause an effective, specific antimicrobial effect against *E. amylovora* and may provide the basis for a novel approach for fire blight control.

## Introduction

Fire blight, caused by the bacterial pathogen *Erwinia amylovora*, is one of the most serious diseases of apple and pear in the United States and worldwide. Fire blight infection can occur in flowers, leaves, shoots, and fruits, resulting in yield reduction; the pathogen also can spread systemically through trees to the rootstock, ultimately resulting in tree death (Norelli et al., 2003; van der Zwet et al., 2012). Annual losses to fire blight and costs of control in the US are estimated at over $100 million (Norelli et al., 2003).

The management of fire blight is challenged due to the availability of limited control options. As the pathogens enter plants through the natural opening of the flowers, antibiotic spray applications during bloom are the most effective and widely used control method for fire blight in the US (Norelli et al., 2003; Sundin et al., 2016). Streptomycin is the most effective antibiotic targeting *E. amylovora*, and has been used for fire blight management in the US since the 1950s (Goodman, 1959). The intensive, long-term use of streptomycin, however, has resulted in the development of streptomycin resistance in the *E. amylovora* population. Since its original report in California in 1972 (Miller and Schroth, 1972), streptomycin resistance has been observed in most major apple-producing regions in the United States (Chiou and Jones, 1995; Evans, 2007; Russo et al., 2008; McGhee et al., 2011). In addition, agricultural application of streptomycin also raises significant concerns for the potential selection of antibiotic resistant bacteria in the environment, and the potential impact to human health (Sundin and Bender, 1996). Besides antibiotics, copper bactericides and other biological control products are also used for fire blight management, however the use of these materials is limited by their inconsistent control efficacy and copper use can also result in phytotoxicity (Tsiantos et al., 2003; Sundin et al., 2009; Johnson and Temple, 2013). Because of these reasons, developing effective control alternatives for fire blight has become an urgent need for sustainable apple and pear production in the US (Khokhani et al., 2013; Yang et al., 2014; Sundin et al., 2016).

RNA silencing is the translational repression of a mRNA caused by the binding of an antisense RNA (Nakashima et al., 2012). In principle, the translation of any mRNA could be silenced by an antisense RNA with sequences complementary to the translational initiation sequences of the target mRNA (Good and Nielsen, 1998; Bennett and Swayze, 2010). The discovery of RNA silencing provides a powerful tool to artificially modulate gene expression. One application of RNA silencing is the synthesis of artificial RNA homologs to silence the expression of essential genes in microbes, and use of these compounds as antimicrobials (Rasmussen et al., 2007).

Antisense antimicrobials are short oligomers of nucleic acid homologs with antisense sequences to the translational initiation sites of essential genes of bacteria (Rasmussen et al., 2007; Bai and Luo, 2012). The binding of the antisense compounds to the translational initiation sites can lead to the silencing of these essential genes and subsequent growth inhibition (Bai and Luo, 2012). Compared to traditional antibiotics, antisense antimicrobials have many unique advantages including: (1) Unlike antibiotics that usually target a universal cellular process and kill bacteria with little selection, antisense antimicrobials can target a specific DNA sequence of the pathogen without affecting the survival of other potentially beneficial, environmental bacteria; (2) Unlike antibiotics that are typically limited to targeting a single cellular process, antisense antimicrobials can target any essential genes through sequence complementation, thus significantly enlarging the target selection; (3) In the case of a pathogen developing resistance to antisense antimicrobials through mutations, the resistance could be overcome by designing new antisense sequences against the mutated sequence.

The fact that DNA and RNA are unstable under UV and can be easily degraded by enzymes makes them undesirable materials for antisense antimicrobials. Improvements designed to modify or replace the sugar-phosphate backbone of DNA/RNA has resulted in nucleic acid homologs with significantly enhanced stability (Bai and Luo, 2012). These nucleic acid homologs include peptide nucleic acids (PNAs), phosphorodiamidate morpholino oligomers (PMOs), and phosphorothioate oligonucleotides (PS-ODNs) (Bai and Luo, 2012). Among them, PNAs have shown promising antimicrobial effects against some animal pathogenic bacterial species (Good and Nielsen, 1998; Nekhotiaeva et al., 2004; Kulyte et al., 2005; Kurupati et al., 2007; Hatamoto et al., 2010). In PNA, the sugar–phosphate backbone of DNA/RNA was replaced with a pseudopeptide backbone, while nearly identical geometry and spacing of the bases was retained (Bai and Luo, 2012). This modification significantly enhances the stability, as the PNA molecules are stable when exposed to bacterial cytoplasmic extracts, human blood serum, and enzymes that degrade DNA and peptide (DNase, protease, et al.) (Demidov et al., 1994; Bai and Luo, 2012). In addition, it also increases the PNA–RNA binding affinity, as RNA is negatively charged but PNA is electrically neutral (Bai and Luo, 2012). However, since PNA oligomers are large molecules, they may not enter the cellular membrane as readily. Thus, the delivery of PNA oligomers into bacterial cells often requires external assistance. Cell penetrating peptides (CPPs) are short peptides of less than 30 amino acids that can penetrate cell membranes and deliver covalently conjugated cargoes into cells (Abes et al., 2007; Bai and Luo, 2012). Conjugation of CPPs with PNAs has been demonstrated to significantly increase PNA entry into bacterial cells (Zatsepin et al., 2005; Rasmussen et al., 2007). Two features of CPPs that are required for cell penetrating ability include amphipathicity and positive charge (Wu et al., 2007; Bai and Luo, 2012). These characteristics are acquired by synthesizing CPPs with an alternation of cationic amino acid residues and nonpolar residues. No obvious toxicity of CPP to animal cells has been observed and CPP is considered safe to use as drug delivery in human medicine (Dinca et al., 2016).

PNA–CPPs have been successfully used as antisense antimicrobials in both *in vitro* and *in vivo* trials against animal pathogenic bacteria. For example, CPP-conjugated PNAs targeting essential genes such as *acpP, inhA, gyrA, ompA*, 16 s rRNA, and *adk* have shown significant growth inhibition effect against a number of bacteria including *Escherichia coli* (Good and Nielsen, 1998; Tan et al., 2005), *Pseudomonas aeruginosa* (Ghosal and Nielsen, 2012), *Staphylococcus aureus* (Hatamoto et al., 2010), *Mycobacterium smegmatis* (Kulyte et al., 2005), and *Klebsiella pneumoniae* (Kurupati et al., 2007). Previous research suggests that the start codon and Shine-Dalgarno (SD) region of the mRNA are the most sensitive sequences for inhibition caused by antisense antimicrobials (Dryselius et al., 2003).

Although PNA–CPPs have shown some promising applications in controlling bacterial infections in animal models, to our knowledge, no research has explored the use of PNA–CPP in controlling plant diseases. Regulatory small RNAs (sRNAs) play important roles in modulating gene expressions in bacteria (Gottesman and Storz, 2010). We previously have identified regulatory small RNAs in the fire blight pathogen *E. amylovora* and have described the roles of these sRNAs in regulating various virulence and cellular functions (Zeng et al., 2013; Zeng and Sundin, 2014). These findings suggest that RNA silencing is a naturally occurring process in *E. amylovora* and that it is possible that the expression of a given gene could also be modulated by artificially synthesized RNA homologs. In this research, we explored the proof-of-concept of using PNA–CPP that targets an essential gene *acpP* in *E. amylovora* as an antimicrobial, and using this compound to control fire blight. We determined the application conditions (selection of essential genes and CPP), mechanisms of antimicrobial activity, and documented effectiveness of PNA–CPP in limiting pathogen growth on detached apple flowers.

## Materials and Methods

### Bacterial Strains, Culture Conditions, and PNA–CPP Synthesis

The highly virulent strain *E. amylovora* Ea110, which was isolated from an apple orchard in Michigan (Zhao et al., 2005), was used in this study. Bacterial strains were stored at -80°C in 15% glycerol and cultured in Luria–Bertani (LB) medium at 28°C. PNA–CPP was synthesized using Bts oligomerization method by Panagene Inc (Daejeon, Korea). Streptomycin and PNA–CPP were implemented at rates indicated in each assay.

### Measurement of Bacterial Growth Inhibition

*Erwinia amylovora* Ea110 was cultured in LB broth overnight and then cell concentrations were adjusted to 5 × 10^5^ CFU/ml in LB broth. A total of 80 μl of the bacterial suspension in LB was added into each well of a 96-well plate. Lyophilized PNA–CPP was resuspended in water to a stock concentration of 100 μM and was serial diluted. Twenty microliter of the diluted PNA–CPP solution was added into each well that contained 80 μl of the bacterial suspension in LB to make the final concentrations indicated in each assay. Water and streptomycin were added to the wells as negative and positive controls. The plate was incubated at 28°C with orbital shaking in a BioTek Synergy H1 microplate reader (BioTek, Winooski, VT, USA) for 20 h. During the incubation, the OD 600 of each well was measured every 10 min. The lowest concentration of a PNA–CPP that prevented growth after 20 h represented the MIC. Three replicates were included in each testing, and the experiment was repeated twice.

### Viability Test

Overnight cultures of *E. amylovora* Ea110 were adjusted to 5 × 10^5^ CFU / ml in LB broth. Forty microliters of anti-*acpP-*CPP1 (100 μM), streptomycin (100 μM), or H_2_O were added into 460 μl of the LB broth containing *E. amylovora* cells to reach a final concentration of 8 μM of the compounds mentioned above. The cultures were incubated at 28°C with 200 rpm of continuous shaking. Samples were taken at 0, 1, 2, 3, 4, 5, and 6 hours post inoculation. Bacterial cells from each sample were collected by centrifugation (6500 rpm for 8 min), washed with sterile water to remove the residual compounds, diluted 10^2^ to 10^5^-fold, and plated on LB agar plates. Colonies formed after 48 hour-incubation at 28°C are counted and original cell concentration was calculated. Five replicates were included in each assay and the experiment was repeated twice with similar results observed.

### Fluorescence Microscopy

Bacterial cells were washed with sterile water to remove residual medium from the culture. LIVE/DEAD BacLight bacterial viability kit (Molecular Probes, Eugene, OR, USA) components A and B were added to the cells following the manufacturer’s instructions. Green fluorescence and red fluorescence of cells stained were observed using a Zeiss Scope A1 fluorescence microscope equipped with a FITC filter and a DAPI filter (Oberkochen, Germany). Images were acquired with a Spot RT3 camera using the Spot Advanced software (Sterling Heights, MI, USA). Statistical analysis was performed using ANOVA with α = 0.05. The level of significance labeled in figure (denoted by different letters) was calculated using LSD (α = 0.05). ANOVA and LSD analyses were conducted in R ver. 3.3.1 (R Core Team, 2015).

### RNA Isolation

*Erwinia amylovora* Ea110 was inoculated into LB broth at the final concentration of 5 × 10^5^ CFU/ml, and was treated with various concentrations of PNA–CPP, water, or streptomycin. The inoculated cells were incubated at 28°C with orbital shaking, and were collected for RNA isolation at 15.5 h post inoculation. Total bacterial RNA was isolated using the RNeasy Protect Bacteria Mini Kit (Qiagen, Valencia, CA, USA) following the manufacturer’s instructions. The quality and quantity of RNA was measured with a Nanodrop 2000c (Thermo Scientific, Wilmington, DE, USA).

### Northern Blot

The probe for *acpP* detection in Northern blots was synthesized from polymerase chain reaction (PCR) using *acpP* primers (forward primer 5′-TGG GCG TTA AGC AGG AAG AAG-3′ and reverse primer 5′-TAC GCC TGG TGA CCA TTG AT-3′). The PCR product was purified using a QIAquick PCR Purification kit (Qiagen) and labeled using a Biotin DecaLabel DNA Labeling Kit (Thermo Fisher Scientific, Grand Island, NY, USA) as per instruction of kit manual. Thirty micrograms of total RNA from different treatments were loaded to a formaldehyde denaturing gel in MOPS buffer. 16s rRNA was quantified using a transluminator as internal control of RNA quantity. BrightStar^®^-Plus positively charged nylon membranes (Thermo Fisher Scientific) were used for transfer of RNA from gel to membrane. Transfer, pre-hybridization, and hybridization were performed following the manufacturer’s instructions of the Northern Max kit (Thermo Fisher Scientific). The biotin signal was detected using a Biotin Chromogenic Detection Kit (Thermo Fisher Scientific). The band intensity was quantified by ImageJ (Schneider et al., 2012).

### Detached Apple Flower Assay

Freshly opened flowers were collected from apple trees *Malus* × *domestica* ‘McIntosh’ from the Connecticut Agricultural Experiment Station Lockwood farm in Hamden, CT, USA) in May 2016. The pedicel of the flowers was detached from the flower cluster, and each flower was placed in a 7 ml plastic tube (containing 10% sucrose solution) through a hole created in the plastic cap. One microliter of *E. amylovora* (Ea110) at the concentration of 10^7^ CFU/ml was inoculated onto the five stigmas of each flower (approximately 0.2 μl per stigma). Twenty microliters of the PNA–CPP at different concentrations were evenly applied onto the stigmas of each flower 2 h before and 19 h after the inoculation. Water and streptomycin (100 μM) were used as negative and positive controls. In the PNA–bacteria mix treatment, PNA at the concentration 20 μM was mixed with an equal volume of *E. amylovora* cells at the concentration of 10^7^ CFU/ml in a microcentrifuge tube and incubated for 30 min at 22–25°C. Two microliters of the mixture were applied evenly onto the five stigmas of the flowers. The pathogen population on the apple stigmas was quantified at 42 h post inoculation, by dissecting the stigmas from the flowers, and suspending them in 1 ml of 0.5x PBS. A Taqman probe realtime PCR assay was used to quantify the pathogen amount. Primers used in this assay are: amsk120F: 5′-CAT GCA ATT TCC AGT TTC CT-3′; amsk120R: 5′-GCA TGA CGG TTA ACC AAA TC-3′, amsk120Probe: 5′-TGC GTG ACC TGA TTC AGC ACA A-3′. The reaction was performed on a Biorad CFX96 Realtime PCR machine. The cell concentrations were calculated by f (Cq) = –3.47 Cq + 45.513. A standard curve was generated using five predetermined concentrations of Ea110 (10^7^, 10^6^, 10^5^, 10^4^, 10^3^ CFU/ml) cells. Six flowers were used in each treatment. The experiment was repeated twice with similar results observed, and results from one representative experiment are displayed. The box plot was generated by R ver. 3.3.1 (R Core Team, 2015). Statistical analysis was performed using ANOVA with α = 0.05. The level of significance labeled in figure (denoted by different letters) was calculated using LSD (α = 0.05). ANOVA and LSD analyses were conducted in R ver. 3.3.1 (R Core Team, 2015).

### Collection of Apple Flower-associated Bacteria

Fifty flowers were collected from apple trees *Malus* × *domestica* ‘McIntosh’ from the Hamden, CT Lockwood farm in May 2016. The stigmas from all 50 flowers were removed, placed into 3 ml of 0.5x PBS, vortexed for 20 s, and sonicated for 5 min in a water bath sonicator to release the stigma associated bacteria. PBS buffer containing bacteria cells was serially diluted (10x, 100x, and 1000x) and plated on LB agar medium and incubated at 28°C for 48 h. On the basis of morphological colony characteristics, 200 bacterial colonies were shortlisted, sub-cultured, and identified using16s rRNA sequencing using the forward primer 63f (5′-CAG GCC TAA CAC ATG CAA GTC-3′) and reverse primer 1387r (5′-GGG CGG WGT GTA CAA GGC-3′). Fifteen different species were selected from the 200 bacterial cultures for testing against anti-*acpP*-CPP1 and streptomycin. Biological control agents *Pseudomonas fluorescence* A506, *Bacillus amyloliquefaciens* D747 and *Pantoea agglomerans* E325 were isolated from the original products on LB agar plate.

## Results

### PNAs with Antisense Sequence to the Start Codon Region of *acpP* Caused Growth Inhibition of *E. amylovora* When Conjugated with CPP

**FIGURE 1:**
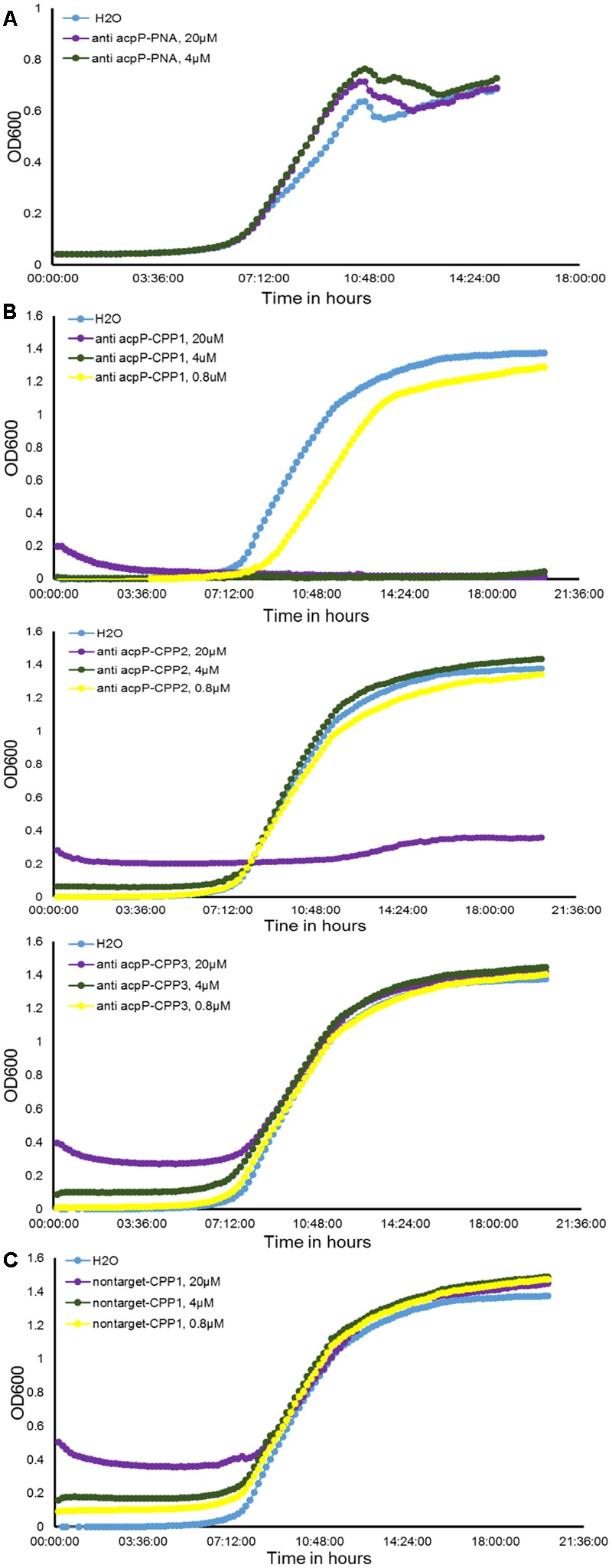
**Effect of peptide nucleic acid–cell penetrating peptide (PNA–CPP) on the growth of *E. amylovora* Ea110 in LB broth. (A)** *Erwinia amylovora* treated with anti-*acpP* PNA. **(B)** *E. amylovora* treated with CPP conjugated PNA: anti-*acpP-*CPP1, anti-*acpP-*CPP2, and anti-*acpP-*CPP3. **(C)** *E. amylovora* treated with CPP1 conjugated PNA containing random sequence (non-target-CPP1). *E. amylovora* Ea110 cells were maintained in 100 μl of LB broth supplemented with PNA–CPPs at different concentrations in a 96-well plate. The 96-well plate was maintained at 28°C with orbital shaking in a plate reader. Turbidity (OD_600_) was measured every 10 min for a period of 20 h. Three replicates were included in the assay and the experiment was repeated twice. Results from one experiment are displayed in this figure.

To explore the possibility of using PNAs as antimicrobials against the fire blight pathogen *E. amylovora*, we first synthesized a 10-nucleotide PNA oligomer with antisense sequence to the start codon region (–5 to +5) of a previously discovered essential gene *acpP* (encoding an acyl carrier protein), anti-*acpP* PNA (**Table 1**) (Zhang and Cronan, 1998). The effect of the anti-*acpP* PNA on the growth of *E. amylovora* was evaluated under an *in vitro* condition. Compared to the water-treated cells, cells treated with anti-*acpP* PNA did not display any growth inhibition at either concentration tested (**Figure 1A**).

**Table 1:** Peptide nucleic acid (PNA)–cell penetrating peptide (CPP) molecules used in this study.

Compound name	Sequence
Anti-*acpP* PNA	**5′- ctcatactat -3′**
Anti-*acpP*-CPP1	(KFF)3K- eg1**-ctcatactat -3′**
Anti-*acpP*-CPP2	**5′- ctcatactat -**BX(RXR)4
Anti-*acpP*-CPP3	YARVRRRGPRGYARVRRRGPRRC- **ctcatactat -3′**
Nontarget PNA–CPP1	(KFF)3K-eg1**-accatggtgg-3′**

*Bold letters indicate PNA sequence, in the 5′ to 3′ order.*

As the ineffectiveness of the anti-*acpP* PNA on *E. amylovora* growth could be due to the inefficient entry of PNA into the cytoplasm, we determined whether conjugation of the anti-*acpP* PNA with CPP would result in enhanced antimicrobial effect. Three formulations of CPP (CPP1, CPP2, and CPP3) that were demonstrated to have promising delivery efficiency of PNAs in animal pathogenic bacteria were then individually conjugated to the anti-*acpP* PNA, resulting in anti-*acpP-*CPP1, anti-*acpP-*CPP2, and anti-*acpP-*CPP3 (**Table 1**). The effect of the CPP-conjugated PNAs on *E. amylovora* growth was tested under the same conditions. Compared to the water control, addition of anti-*acpP*-CPP1 or anti-*acpP*-CPP2 both resulted in complete growth inhibition of *E. amylovora* at the 20 μM concentration, whereas addition of anti-*acpP*-CPP3 did not cause any growth inhibition at any concentrations (**Figure 1B**). The growth inhibition caused by anti-*acpP*-CPP1 was more potent than the inhibition caused by anti-*acpP*-CPP2, suggesting that CPP1 [(KFF)3K] was the most efficient CPP in delivering PNA into *E. amylovora*. To determine if the growth inhibition caused by PNA–CPP was through specific targeting of *acpP*, a PNA with random nucleotide sequence in conjugation with CPP1 (nontarget PNA–CPP1) was also tested (**Table 1**). The nontarget-CPP1 did not cause any inhibition of *E. amylovora* growth (**Figure 1C**).

**FIGURE 2:**
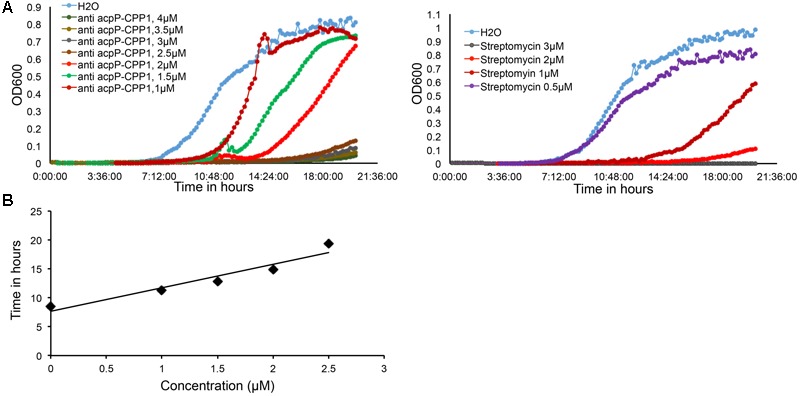
**Dose dependent growth inhibition of *E. amylovora* Ea110 caused by anti-*acpP*-CPP1. (A)** *E. amylovora* Ea110 growth in LB broth in the presence of different concentrations of anti-*acpP*-CPP1 and streptomycin. **(B)** Time to reach OD_600_ = 0.1 of Ea110 growth at different concentrations of anti-*acpP*-CPP1.

We further determined that the minimal inhibitory concentration (MIC) of anti-*acpP*-CPP1 and streptomycin was 2.5 and 2 μM, respectively (**Figure 2A**). In addition, we showed that the growth inhibition caused by anti-*acpP*-CPP1 is positively correlated with the concentrations of anti-*acpP*-CPP1, in a linear manner (**Figure 2B**). Taken together, these results suggest that CPP-conjugated PNA with antisense sequence targeting the start codon of *acpP* is able to cause potent growth inhibition of *E. amylovora* under *in vitro* conditions. This inhibition was caused by the antisense sequence of *acpP* on PNA. CPP was essential in delivering PNA into *E. amylovora* and causing the antimicrobial effect. Among the three CPPs previously used in animal pathogens, CPP1 was the most efficient for PNA delivery into *E. amylovora*.

### *Erwinia amylovora* Cells Treated With Anti-*acpP-*CPP1 Showed Reduced *acpP* mRNA Levels

**FIGURE 3:**
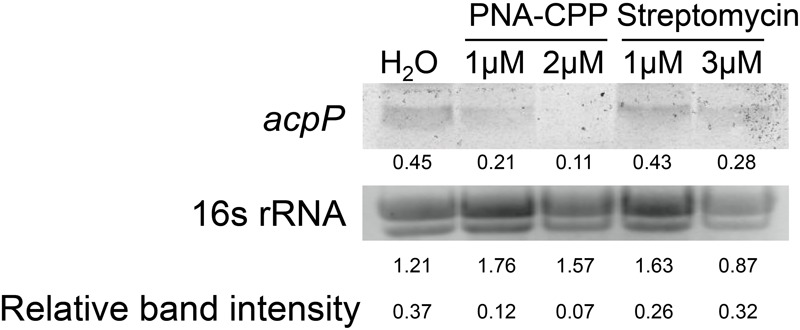
**Northern blot detection of the expression levels of *acpP* in *E. amylovora* Ea110 treated with anti-*acpP-*CPP1 and streptomycin.** *E. amylovora* was cultured in LB broth supplemented with water, anti-*acpP*-CPP1 (at final concentrations 1 and 2 μM), and streptomycin (at final concentrations 1 and 3 μM) for 18 h. Total RNA was quantified by Nanodrop. Thirty micrograms of RNA were loaded to a denaturing gel and analyzed for the *acpP* mRNA amount using a Northern blot assay. The image of 16s rRNA was taken using a transluminator as internal control of RNA quantity between samples. The band intensity was quantified by ImageJ. Relative band intensity was calculated by deviding the intensity of the *acpP* band with the intensity of the 16S band.

After entering the bacterial cytoplasm, PNA–CPP is believed to bind to the start codon region of the target mRNA and block its translation (Good and Nielsen, 1998; Wang and Xu, 2004). However, whether the PNA–mRNA binding would also cause the degradation of the target mRNA is not clear. We compared the levels of *acpP* mRNA in *E. amylovora* cells treated with water, anti-*acpP*-CPP1 at two sub-lethal concentrations (1 and 2 μM), and streptomycin at sub-lethal concentrations (1 and 3 μM) using Northern blot. Compared to the water-treated cells, cells treated with anti-*acpP*-CPP1 showed a visual reduction in the level of *acpP* mRNA. This reduction is positively correlated with anti-*acpP*-CPP1 concentrations (**Figure 3**). In contrast, streptomycin treatment did not affect the *acpP* mRNA abundance. These observations suggest that anti-*acpP-*CPP1 binding to *acpP* mRNA caused the degradation of *acpP* mRNA. It also indicates that the growth inhibition caused by anti-*acpP*-CPP1 is through a gene specific interaction with *acpP* mRNA.

### The Growth Inhibition of *E. amylovora* by Anti-*acpP-*CPP1 Is Bactericidal

**FIGURE 4:**
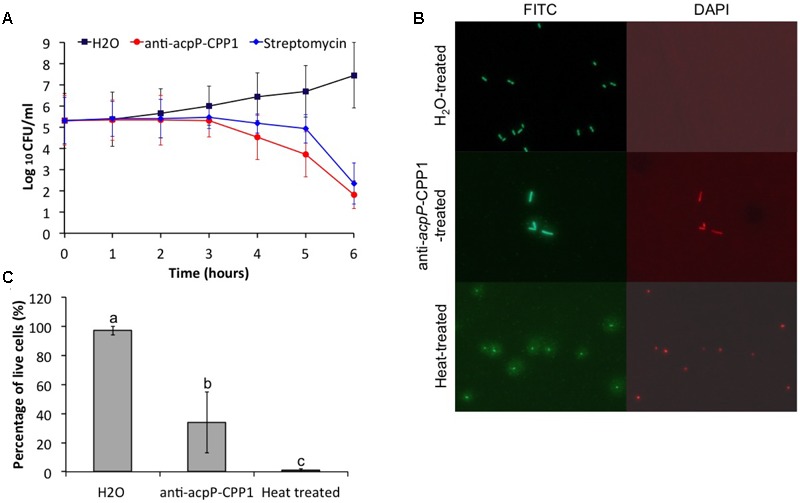
**Effect of anti-*acpP*-CPP1 on the viability of *E. amylovora* Ea110. (A)** Number of viable *E. amylovora* cells recovered on LB plates from the anti-*acpP*-CPP1 and streptomycin treatment. **(B)** *E. amylovora* stained with fluorescent dye SYTO 9 and propidium after 6 hrs of treatment with anti-*acpP*-CPP1 and water. Heat-treated cells (80°C for 10 mins) were included as a control. **(C)** Percentage of live cells of the total *E. amylovora* cells after 6 hrs of treatment with water, anti-*acpP*-CPP1, and treated with heat (80°C for 10 mins). *E. amylvoora* Ea110 (5 × 10^5^) were inoculated into LB broth supplemented with anti-*acpP*-CPP1 or water, and incubated at 28°C with shaking. The viability of *E. amylovora* cells was tested at different time points by plating on LB agar plates and by fluorescence staining and microscopy. Statistical analysis was performed using ANOVA with α = 0.05. The level of significance was calculated using LSD (α = 0.05).

To determine whether the growth inhibition of *E. amylovora* by anti-*acpP-*CPP1 was bactericidal or bacteriostatic, we tested the viability of *E. amylovora* cells in LB broth at different time points after treatment of anti-*acpP-*CPP1 (8 μM), streptomycin (8 μM), or water, using a serial dilution plating method. In the water-treated sample, we observed an exponential increase in the amount of viable cells after inoculation (**Figure 4A**). Following treatment with anti-*acpP-*CPP1, the viable cell count remained the same during the first 3 h, displaying a bacteriostatic effect (**Figure 4A**). After 3 h, the viability of cells treated with anti-*acpP-*CPP1 drastically declined, which suggested that anti-*acpP-*CPP1 caused a bactericidal effect during this period. After 6 h of treatment with anti-*acpP-*CPP1, the number of viable cells was reduced by more than 1000-fold (log_10_ CFU/ ml reduced from 5.3 at 0 h to 1.8 at 6 h, **Figure 4A**), at a slightly faster rate than streptomycin. These observations suggest that the growth inhibition of *E. amylovora* caused by anti-*acpP*-CPP1 is bactericidal, similar to the traditional antibiotic streptomycin.

The mechanism of the growth inhibition caused by anti-*acpP*-CPP1 was also studied using a bacterial viability kit and fluorescence microscopy. At 6 h after inoculation, cells treated with water were green fluorescent but not red fluorescent when stained with a mixture of fluorescent dye SYTO 9 and propidium iodide, suggesting that these cells were viable (**Figure 4B**). However, a large number of cells treated with anti-*acpP-*CPP1 at a concentration above the MIC (8 μM) were both green fluorescent and red fluorescent, suggesting that many of these cells lost viability (**Figures 4B,C**). Interestingly, cells treated with anti-*acpP*-CPP1 showed an elongated cell shape compared to the water-treated cells (**Figure 4B**). No cell lysis (as shown in the heat-treated cells in **Figure 4B**) was observed following treatment with anti-*acpP*-CPP1, which suggests that the bactericidal effect may not be caused by physical disintegration of the cell structure (**Figure 4B**). The fluorescence microscopy observation is consistent with the plating method, together suggesting that the growth inhibition of *E. amylovora* caused by anti-*acpP*-CPP1 is bactericidal.

### Anti-*acpP-*CPP1 was Able to Inhibit Pathogen Growth on Apple Stigmas

**FIGURE 5:**
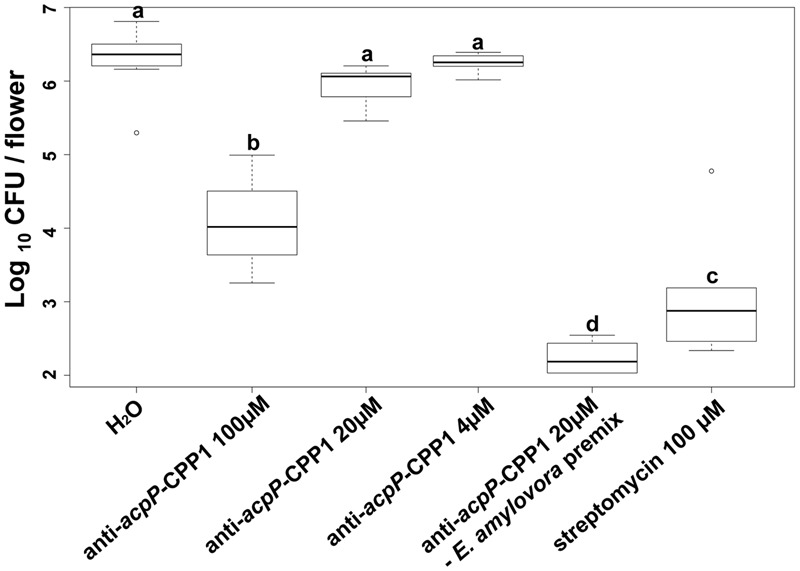
**Amount of *E. amylovora* Ea110 cells on detached apple stigmas after treatment with anti-*acpP*-CPP1 and streptomycin.** *E. amylovora* Ea110 cells (10^7^ CFU/ml) were inoculated on the stigma of freshly opened ‘McIntosh’ flowers (1 μl per flower by a pipette). Twenty microliters of anti-*acpP*-CPP1 (100, 20, and 4 μM) were evenly applied onto the stigmas of each flower 2 h before and 19 h after the inoculation. Water and streptomycin (100 μM) were used as negative and positive controls. In the PNA–bacteria mix treatment, anti-*acpP*-CPP1 (20 μM) was mixed with an equal volume of *E. amylovora* cells (10^7^ CFU/ml) in a microcentrifuge tube and incubated for 30 min at room temperature (22–25°C). Two microliters of the mixture were applied evenly onto the five stigmas of the flowers. All flowers were incubated at room temperature and the pathogen population on the apple stigmas was quantified at 42 h post inoculation, using a Taqman probe realtime PCR assay. Statistical analysis was performed using ANOVA with α = 0.05. The level of significance (denoted by different letters) was calculated using LSD (α = 0.05).

Following demonstration that anti-*acpP*-CPP1 has potent growth inhibition of *E. amylovora* under *in vitro* conditions, we further evaluated the effectiveness of anti-*acpP*-CPP1 in inhibiting *E. amylovora* growth on apple stigmas using a detached flower assay. Compared to the water-treated control, stigmas treated with 100 μM anti-*acpP-*CPP1 showed a significant reduction of *E. amylovora* population (>100-fold reduction, **Figure 5**). However, this reduction was less potent in comparison to the reduction by streptomycin (**Figure 5**). At lower concentrations, anti-*acpP-*CPP1 treatment did not result in significant reduction of the pathogen population when directly applied to the apple stigmas (**Figure 5**). However, premixing anti-*acpP-*CPP1 (20 μM) with *E. amylovora* cells for 30 min before applying the cell-compound mixture onto the apple stigmas resulted in excellent reductions of pathogen populations on apple stigmas (**Figure 5**). The results above suggest that anti-*acpP-*CPP1 is able to inhibit pathogen growth on apple stigmas when applied at 100 μM. It also suggests that the efficacy of anti-*acpP-*CPP1 may be improved if optimal cell-compound contact can be established.

### Effect of Anti-*acpP-*CPP1 on Microbiome Associated with Apple Flowers

**FIGURE 6:**
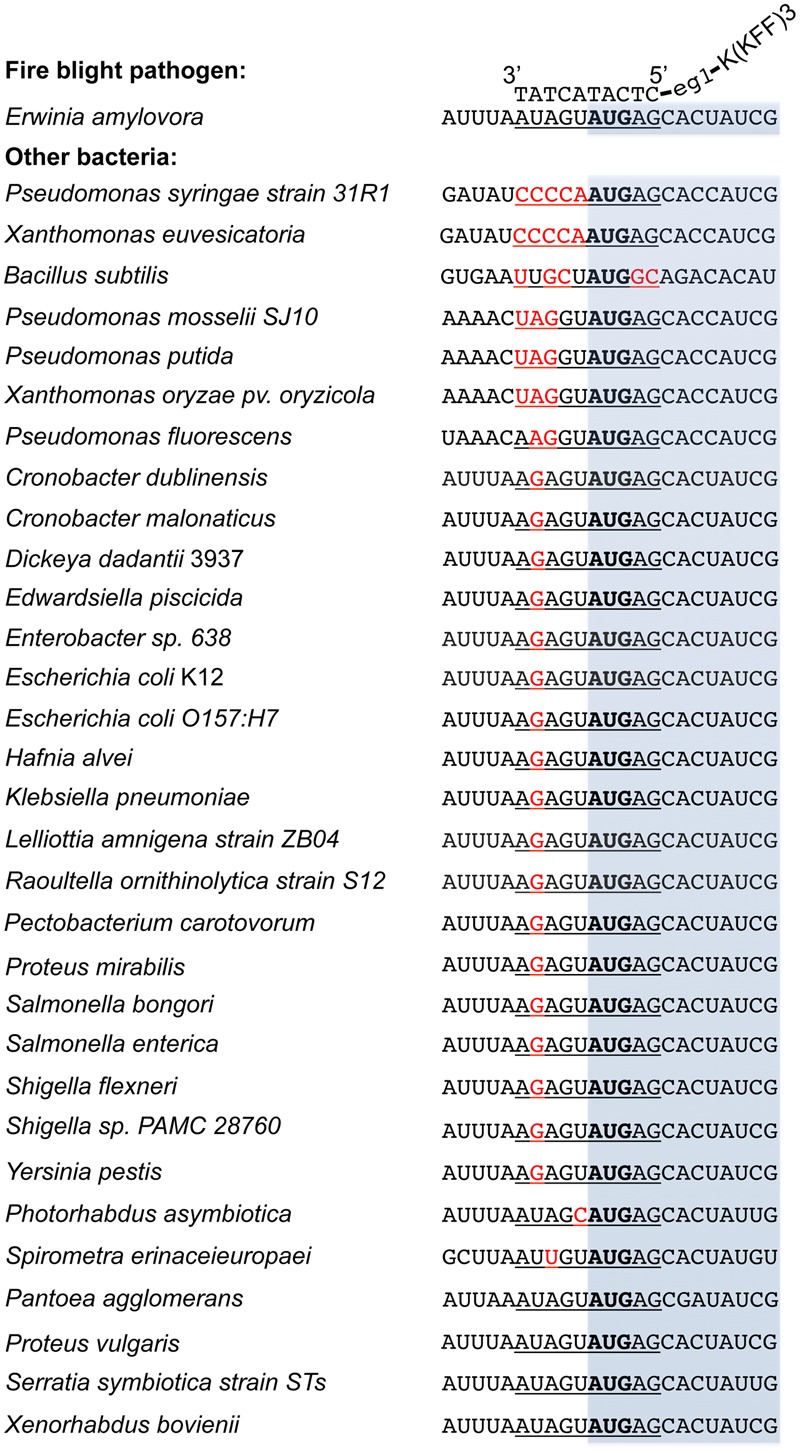
**Alignment of the *acpP* start codon sequences of *E. amylovora* with other bacterial species.** Sequences were obtained from NCBI database based on the availability of the bacterial genomes. The –5 to +5 sequences where the PNA–CPP targets are underlined, with mismatches heighted in red and start codon in bold. The open reading frame of *acpP* was shaded in light blue. The binding of PNA–CPP to *acpP* in *E. amylovora* was also illustrated. All sequences were written in a 5′ to 3′ order otherwise labeled.

Compared to the traditional antibiotics that kill microbes with little selection, antisense antimicrobials inhibit bacterial growth through a sequence specific manner. To determine whether anti-*acpP*-CPP1 affects the growth of environmental microbes associated with apple flowers, bacteria were first cultured from freshly collected ‘McIntosh’ apple flowers. Two hundred bacterial colonies were subcultured, and the identities of these colonies were determined by 16s sequencing. The sequencing and Blast analyses suggest that these bacteria belong to 15 different species (**Table 2**). Representative strains from each species were tested for their susceptibility to anti-*acpP-*CPP1 and streptomycin at concentrations above the MICs for *E. amylovora* (4 and 20 μM, respectively). Our results suggest that anti-*acpP-*CPP1 did not affect the growth of most species (12/15), such as *Pseudomonas* ssp., *Bacillus muralis, Curtobacterium plantarum*, and multiple species in the *Pantoea* genus. However, three species, *P. ananatis, P. agglomerans*, and *Lysobacter oligotrophicus* displayed susceptibility to anti-*acpP-*CPP1 similar to *E. amylovora*. Comparison of the start codon sequences of *acpP* in species with genomes available in NCBI revealed that many species contain sequence discrepancies with *E. amylovora* in this region, although some other species contain similar sequences to *E. amylovora* (**Figure 6**). *P. agglomerans* that has an identical sequence as *E. amylovora* was also susceptible to anti-*acpP-*CPP1. In addition to the bacterial species isolated from the apple flower, we also tested the susceptibility of three microorganisms from fire blight biological control products to anti-*acpP-*CPP1. Similar to the environmental isolates, *Pseudomonas fluorescens* A506 and *Bacillus amyloliquefaciens* D747 (active ingredients of BlightBan A506 and Double Nickel) were not affected by anti-*acpP-*CPP1 whereas *P. agglomerans* E325 (active ingredient of Bloomtime Biological) was susceptible (**Table 2**). All strains tested are susceptible to streptomycin with the exception of *P. fluorescens* A506 (**Table 2**). Finally, some other plant and animal pathogens, such as *Dickeya dadantii* and *Pectobacterium carotovorum* are susceptible to anti-*acpP-*CPP1 (**Table 2**) and further examination of their sequences suggests they have similar sequences targeted by anti-*acpP-*CPP1 as *E. amylovora.*

**Table 2:** The effect of anti-*acpP-*CPP1 and streptomycin.

Bacterial species	Growth in LB broth amended with anti-*acpP*-CPP1 (4 μM)	Growth in LB broth amended with streptomycin (20 μM)	Growth in LB broth
**Environmental isolates recovered from apple flowers^∗^**			
*Pseudomonas savastanoi*	+	–	+
*Pseudomonas syringae*	+	–	+
*Pseudomonas marginalis*	+	–	+
*Pseudomonas trivialis*	+	–	+
*Pseudomonas rhizosphaerae*	+	–	+
*Pseudomonas graminis*	+	–	+
*Pseudomonas simiae*	+	–	+
*Pseudomonas poae*	+	–	+
*Bacillus muralis*	+	–	+
*Pantoea ananatis*	–	–	+
*Pantoea brenneri*	+	–	+
*Pantoea vegans*	+	–	+
*Pantoea agglomerans*	–	–	+
*Curtobacterium plantarum*	+	–	+
*Lysobacter oligotrophicus*	–	–	+
**Bacterial species from commercial biocontrol agents for fire blight**			
*Pantoea agglomerans* E325 (Bloomtime Biological)	–	–	+
*Pseudomonas fluorescence* A506 (BlightBan A506)	+	+	+
*Bacillus amyloliquefaciens* D747 (Double Nickel)	+	–	+
**Other human and plant pathogens**			
*Dickeya dadantii* 3937	–	–	+
*Escherichia coli* K12	–	–	+
*Pectobacterium carotovorum*	–	–	+
*Pseudomonas aeruginosa* PAO1	+	–	+

*^∗^Bacterial species were identified by 16s sequencing. The names of the species are the ones with the highest similarity from the NCBI blast search.*

## Discussion

In this study, we demonstrated the proof-of-concept of using PNA–CPPs as antimicrobials against the fire blight pathogen *E. amylovora.* We showed that a 10-bp oligomer of PNA containing antisense sequence of the essential gene *acpP* is able to cause complete growth inhibition of *E. amylovora* at a similar efficacy of streptomycin under *in vivo* conditions. Although there are two additional sites on the chromosome (in *hrcU* and EAM_0144) that could be potentially bound by the 10-bp oligomer PNA, *hrcU*, and EAM_0144 (encoding a T3SS protein and a fatty acid synthesis regulator) are not known as essential genes in bacteria and reduction of *acpP* mRNA was observed upon treatment of anti-*acpP-*CPP1 (**Figure 3**), which suggests that the growth inhibition of anti-*acpP-*CPP1 should be through the interaction with *acpP* mRNA. We also showed that conjugation of the PNA with a CPP is essential for the PNA to exert antimicrobial effect in *E. amylovora* and identified the most effective CPP sequence for *E. amylovora*. We provided evidence that the antimicrobial effect observed is bactericidal. And finally, no phytotoxicity (e.g., scorching, browning, or any other types of obvious damage) was observed in flowers treated with PNA–CPP molecules only during the flower assay. These qualities suggest that PNA–CPPs are good alternatives for streptomycin in future fire blight management. Conditions generated from this research will add to our knowledge base for the practical development of formulations, rates, and timing for future applications, although many other factors, such as the toxicity and the stability of PNA–CPP in the natural environment, need to be determined before application.

Considerations that are often taken into account when developing antimicrobials include effectiveness, specificity, sustainability, and cost. We demonstrated that anti-*acpP-*CPP1 has a potent antimicrobial effect against *E. amylovora* with higher strain specificity than traditional antibiotics. We hypothesize that the antisense antimicrobials may also have good sustainability: if resistance is developed through mutations, the mutated nucleotide(s) could be identified by sequencing, and new antisense molecules could be synthesized to target the mutated sequence. Some bacteria may be able to generate resistance by acquiring multi-drug transporters. However, it is not very likely in this case as previous work demonstrated that PNA–CPPs cannot be readily transported out of the cells due to the large molecular weight (Nikravesh et al., 2007). Furthermore, as PNA–CPP molecules target individual essential genes rather than a conserved biological process like antibiotics, it widens our selection of targets during the development of antimicrobials. Combining PNA–CPP targeting multiple essential genes in *E. amylovora* may provide more potent and robust growth inhibition, although further actions need to be taken to enhance the entry of PNA–CPP molecules into bacterial cells. Currently, the biggest limitation of agricultural application of PNA–CPP is the cost. With the current synthesis method, the cost of PNA–CPP is about $1000USD/μMol. Thus, research in developing new synthesis technologies and improving the synthesis efficacy is essential for future large-scale application of PNA–CPP.

Prior to this research, the use of PNA–CPPs in controlling bacterial infections has been explored in human medicine. Compared to human medicine application, there are essential differences in agricultural use. First, the delivery method and locations where the antimicrobial activity occurs are different in the two systems. Antimicrobials for human medical purposes are usually delivered through the circulation system and the antimicrobial activity occurs mostly internally in the human body. For agricultural use, the delivery is mostly achieved through aerial spray to the plant surface, and the antimicrobial activity occurs mostly externally on the plant surface. Second, non-pathogenic, beneficial bacteria are not readily used as a disease control strategy in human medicine, but the beneficial, pathogen-antagonistic microbes are of great use in plant disease management as biological controls. This emphasizes a need of selectively targeting the pathogen without affecting the non-pathogenic, beneficial bacteria in agricultural settings.

With the differences between the two systems, our study demonstrated that the application of a PNA–CPP can reduce the bacterial pathogen population on plant surfaces. Our results also emphasized the importance of ensuring the compound-bacteria contact for the optimization of antimicrobial results, as we showed that premixing bacteria with PNA–CPP ensured the full bacteria-compound contact and had the most potent antimicrobial effect. It is possible that the use of a nonionic surfactant may be able to improve the antimicrobial effect of PNA–CPP on plant surfaces. In addition, whether the reduction in pathogen population on stigmas would also result in the reduction in blossom blight infection incidence needs to be further evaluated in future orchard trials. Finally, the stability of PNA–CPP in natural environment, when exposed to UV radiation, rain, and temperature changes, is not known. Understanding the stability will help us formulate the product rates, and timing of sprays to achieve the optimal control efficacy in the field.

Our study also showed that the antimicrobial effect of PNA–CPP molecules is more specific to *E. amylovora* and less toxic to other environmental bacteria than traditional antibiotics. Not affecting the environmental microbiome has many benefits. First, healthy microbiota serves as competitors of the pathogens for nutrients, space and can have a positive effect in preventing and limiting disease occurrence. Second, the selective pressure on the total microbiome of antibiotics often facilitated the development and spread of antibiotic resistance into pathogen population (Sundin and Bender, 1996). For example, the streptomycin resistance genes *strA–strB* that confer streptomycin resistance in the fire blight pathogen *E. amylovora* are believed to be acquired from saprophytic bacteria isolates such as *P. agglomerans* through horizontal gene transfer (Chiou and Jones, 1991). Thus, compared to streptomycin and other antibiotics, PNA–CPP may be a more sustainable management approach for fire blight.

One strategy of reducing the usage of antibiotics in tree fruit disease management is to combine biocontrol agents with antibiotics. However, as antibiotics and biocontrol agents are often non-compatible, the antibiotics and biocontrol agents often have to be applied separately, which not only adds labor cost and time constraints, but also may reduce the efficiency. Here we showed that anti-*acpP-*CPP1 is compatible with multiple biocontrol agents such as *P. fluorescens* and *B. subtilis*. The combined application of biological control agents with PNA–CPP may further enhance the control efficacy.

We observed that PNA–CPP antisense antimicrobials caused degradation of the target mRNA of an essential gene with a subsequent bactericidal effect against *E. amylovora*. This suggests that the antimicrobial effect of PNA–CPP on *E. amylovora* is potent and permanent. It also suggests that besides the potential application in disease management, PNA–CPPs may also be used as an effective approach to modulate gene expression in *E. amylovora* and potentially other bacteria. As the mRNA inhibition and dose of PNA–CPP is positively correlated, gene expression can be easily repressed at different levels for molecular research purposes. Thus, the gene expression repression by PNA–CPPs may have potential advantages over the gene knockout approach. This is specifically true in situations such as studying the functions of essential genes, when the gene knockout approach is not an option.

In summary, we performed the first exploration of using PNA–CPPs in controlling a bacterial plant disease. Plant diseases caused by bacteria have a long history of control difficulties, and the lack of effective management options results in significant economical losses worldwide (Sundin et al., 2016). The results produced from this work suggest that antisense antimicrobials may be a valuable future choice for bacterial plant disease management.

## Author Contributions

RP performed most experiments and analyses. GS and C-HY helped with the experiment design and edited the manuscript. JW, RH, and XY helped with some experiment procedures the analyses. QZ designed this study, performed some experiments, and wrote the manuscript. All authors read and approved the manuscript.

## Conflict of Interest Statement

The authors declare that the research was conducted in the absence of any commercial or financial relationships that could be construed as a potential conflict of interest.
